# Development and validation of a radiopathomics model for predicting liver metastases of colorectal cancer

**DOI:** 10.1007/s00330-024-11198-1

**Published:** 2024-12-02

**Authors:** Han-Hui Jing, Di Hao, Xue-Jun Liu, Ming-Juan Cui, Kui-Jin Xue, Dong-Sheng Wang, Jun-Hao Zhang, Yun Lu, Guang-Ye Tian, Shang-Long Liu

**Affiliations:** 1 Department of Gastrointestinal Surgery, The Affiliated Hospital of Qingdao University, Qingdao, People’s Republic of China; 2 School of Control Science and Engineering, Shandong University, Jinan, People’s Republic of China; 3 Department of Radiology, The Affiliated Hospital of Qingdao University, Qingdao, People’s Republic of China; 4 Department of Gastroenterology, The Affiliated Hospital of Qingdao University, Qingdao, People’s Republic of China

**Keywords:** Radiomics, Colorectal cancer, Liver metastases

## Abstract

**Objective:**

To compare the ability of a model based on CT radiomics features, a model based on clinical data, and a fusion model based on a combination of both radiomics and clinical data to predict the risk of liver metastasis after surgery for colorectal cancer.

**Methods:**

Two hundred and twelve patients with pathologically confirmed colorectal cancer were divided into a training set (*n* = 148) and a validation set (*n* = 64). Radiomics features from the most recent CT scans and clinical data obtained before surgery were extracted. Random forest models were trained to predict tumors with clinical data and evaluated using the area under the receiver-operating characteristic curve (AUC) and other metrics on the validation set.

**Results:**

Fourteen features were selected to establish the radiomics model, which yielded an AUC of 0.751 for the training set and an AUC of 0.714 for the test set. The fusion model, based on a combination of the radiomics signature and clinical data, showed good performance in both the training set (AUC 0.952) and the test set (AUC 0.761).

**Conclusion:**

We have developed a fusion model that integrates radiomics features with clinical data. This fusion model could serve as a non-invasive, reliable, and accurate tool for the preoperative prediction of liver metastases after surgery for colorectal cancer.

**Key Points:**

***Question*** *Can a radiomics and clinical fusion model improve the prediction of liver metastases in colorectal cancer and help optimize clinical decision-making*?

***Findings*** *The presented fusion model combining CT radiomics and clinical data showed superior accuracy in predicting colorectal cancer liver metastases compared to single models*.

***Clinical relevance*** *Our study provides a non-invasive, relatively accurate method for predicting the risk of liver metastasis, improving personalized treatment decisions, and enhancing preoperative planning and prognosis management in colorectal cancer patients*.

## Introduction

Colorectal cancer (CRC) has become the third most common malignancy and the second leading cause of cancer mortality worldwide [1]. The main cause of death is postoperative liver metastases, with at least 50% of patients with CRC developing liver metastases postoperatively or late in the course of the disease [2–5]. The tumor-node-metastasis (TNM) staging system is the tool most widely used for the prediction of liver metastases; however, the clinical outcome is highly variable, even among patients with the same TNM stage [6–8], suggesting that classical TNM staging does not provide accurate or complete predictive information [9–11]. Therefore, there is an urgent need for an accurate classification system for the prediction of liver metastases in patients with CRC.

The gold standard for diagnosis of liver metastases after surgery for CRC is pathological examination. However, in view of the invasiveness of the procedure, it is not feasible to perform it frequently and access after radical resection should not be used as a guide for developing a preoperative treatment strategy [12]. Conventional clinical and computed tomography (CT) data can be used to identify patients at higher risk of distant metastases but require a high level of clinician experience. In radiomics, radiologic images are evaluated using quantitative textural information known as imaging features [13, 14]. Radiologic texture features can determine the prognostic value associated with tumor heterogeneity and metastasis in the tumor microenvironment.

Preoperative treatment helps to decrease the risk of tumor metastasis, and more rapid and accurate disease staging and patient selection may help to reduce this risk further [15]. The aim of this study was to establish non-visual information related to the imaging clinic, which tells the detailed and comprehensive characterization of the tumor phenotype to evaluate postoperative metastases in patients with CRC [16].

## Materials and methods

The study protocol was approved by the institutional review board of the Affiliated Hospital of Qingdao University. The need for written informed consent was waived in view of the retrospective observational nature of the research and the anonymity of the data. Patients with CRC who underwent CT between January 2017 and December 2022 and had baseline clinical data available were enrolled in the study. The inclusion criteria were as follows: histopathologically confirmed CRC; standard CT of the abdomen and pelvis performed before any treatment; clinical data available; and complete CT datasets available. The following exclusion criteria were applied: liver metastases present before radical surgery for CRC; other malignant disease present during the study period; and lack of availability of clinicopathologic or follow-up data.

### Collection of clinical data

Information on age, sex, body mass index (BMI), carcinoembryonic antigen (CEA), carbohydrate antigen 19-9 (CA19-9), albumin (ALB), and alpha-fetoprotein (AFP) levels was obtained from the electronic medical records. Tumor size, T stage, and N stage were determined by two radiographers experienced in imaging patients with CRC.

### Acquisition and preprocessing of CT data

Abdominal or pelvic CT was performed on a multidetector row scanner (SOMATOM Definition Flash, Siemens Medical Systems; iCT 256, Philips Healthcare; or Optima CT670, GE Healthcare). All images were reconstructed using the standard reconstruction kernel, including. CT images saved in DICOM (Digital Imaging and Communications in Medicine) format were retrieved from the PACS (Picture Archiving and Communication System).

### Tumor segmentation

A junior radiologist under the supervision of a senior radiologist manually contoured the tumors slice-by-slice using 3D Slicer software (version 5.2.2, https://www.slicer.org/). All images were then read and scored by the same experienced radiologist. If there is a difference that has always been different, it needs to be revised after discussion between the two radiologists.

### Extraction of radiomics features

We used advanced radiomics feature extraction methods to analyze and decode the imaging phenotypes of CRC lesions. After obtaining CT images of the tumors along with their region of interest (ROI) masks, we applied cubic B-spline interpolation to resample the CT images. Given that the ROI masks are binary images, we used the nearest-neighbor interpolation algorithm to resample the mask images. A new imaging spacing of [1 mm, 1 mm, 1 mm] was obtained. All operations adhered strictly to the International Biomarker Standardization Initiative guidelines to ensure the scientific rigor and reproducibility of the feature extraction process.

We used a comprehensive feature selection approach that combined *t*-tests and L1 regularization based on logistic regression to optimize the set of radiomics features for the diagnosis model of CRC. Before selecting the features, we conducted a test for homogeneity of variance. Features with homogeneity of variance (*p* > 0.05) were initially screened with *t*-tests to ensure comparability among the sample features. Otherwise, Welch’s test was used as a correction to the *t*-test to identify features that showed significant differences between negative and positive samples.

Next, we applied further L1 regularization by compressing the weights of unimportant features to zero to reduce the number of unnecessary features and retain the most discriminative features. A logistic regression linear model with the SAGA solver was used as the feature selector, with an L1 penalty term set. The optimal solution among multiple regularization strengths was found using the grid search method.

### Development of the clinical and radiomics models

Clinical and pathological characteristics were used to develop a clinical model for predicting the risk of liver metastasis after surgery for CRC. Initially, we used a *t*-test to screen for clinical features with significant differences. Next, the entire feature set was standardized according to the normalization pattern of the training set. Finally, the random forest classifier was applied to the selected radiomics features and to the clinicopathologic features to independently construct the clinical classification model and the radiomics classification model.

### Evaluation of the models

A flowchart summarizing the proposed prediction model is shown in Fig. S1. To increase the predictive performance, we developed a model that combined CT radiomics and clinical features. We used a score-level fusion approach, merging the predictive scores generated by the CT radiomics model and those generated by the clinical model. Three score fusion strategies (i.e., minimum score fusion, maximum score fusion, and weighted score fusion) were used to construct the fusion model that combined the CT radiomics and clinical models. The predictive scores for the minimum and maximum score fusion strategies were determined by comparing the scores generated by the CT radiomics model with those generated by the clinical model for each case, selecting either the minimum or maximum score. The predictive score in the weighted score fusion strategy was calculated as the weighted sum of the predictive scores from the CT radiomics model and the clinical model, where the sum of the weights of both models was equal to one.

### Statistical analysis

Continuous variables are presented as the mean ± standard deviation and were analyzed using the Mann–Whitney *U*-test or student’s *t*-test. Categorical variables are reported as the number and were analyzed using the chi-squared test or Fisher’s exact test. Delong’s test was used to evaluate the statistical significance of differences between the areas under the curve (AUCs). All data were analyzed using Python (Python Software Foundation) and R Studio (R Foundation for Statistical Computing). A *p*-value < 0.05 was considered statistically significant.

## Results

### Clinical and baseline characteristics

**Fig. 1:**
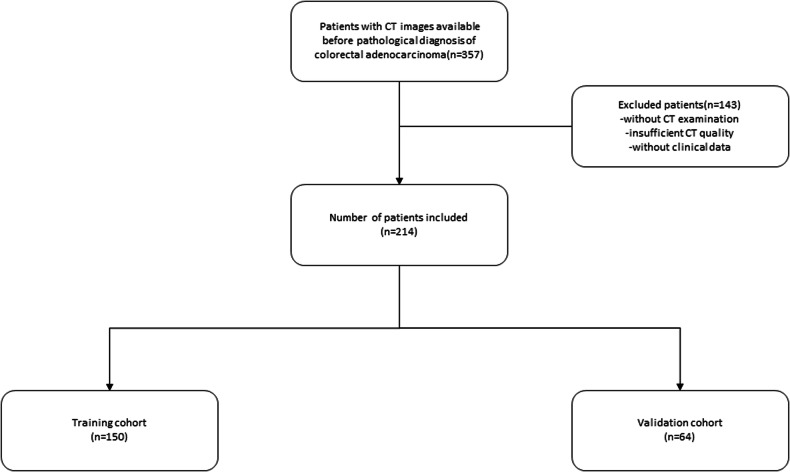
The flowchart of patient inclusion. CT, computed tomography

A total of 212 patients who underwent surgery for CRC between January 2017 and December 2022 were retrospectively included in the study (Fig. 1) and separated into a training set (*n* = 148) and a validation set (*n* = 64). The baseline characteristics of the patients are shown in Table 1.

**Table 1:** Clinical characteristics of patients

Variables	Total, (*n* = 212)	Training cohort, (*n* = 148)	Validation cohort, (*n* = 64)	*p*
Year, mean ± SD	62.24 ± 10.51	61.70 ± 10.88	63.47 ± 9.57	0.262
BMI, mean ± SD	24.3 ± 3.34	24.28 ± 3.27	24.37 ± 3.52	0.859
Sex, (%)				0.981
Male	146 (68.87)	102 (68.92)	44 (68.75)	
Female	66 (31.13)	46 (31.08)	20 (31.25)	
T stage, (%)				0.096
1	3 (1.42)	3 (2.03)	0 (0.00)	
2	21 (9.91)	19 (12.84)	2 (3.12)	
3	144 (67.92)	97 (65.54)	47 (73.44)	
4	44 (20.75)	29 (19.59)	15 (23.44)	
N stage, (%)				0.961
0	93 (43.87)	64 (43.24)	29 (45.31)	
1	68 (32.08)	47 (31.76)	21 (32.81)	
2	50 (23.58)	36 (24.32)	14 (21.88)	
3	1 (0.47)	1 (0.68)	0 (0.00)	
CEA, (%)				0.562
Normal	119 (56.13)	85 (57.43)	34 (53.12)	
Elevated	93 (43.87)	63 (42.57)	30 (46.88)	
CA19-9, (%)				0.082
Normal	168 (79.25)	122 (82.43)	46 (71.88)	
Elevated	44 (20.75)	26 (17.57)	18 (28.12)	
AFP, (%)				0.734
Normal	202 (95.28)	142 (95.95)	60 (93.75)	
Elevated	10 (4.72)	6 (4.05)	4 (6.25)	
ALB, (%)				0.651
Low	101 (47.64)	69 (46.62)	32 (50.00)	
Normal	111 (52.36)	79 (53.38)	32 (50.00)	

*BMI* body mass index, *CEA* carcinoembryonic antigen, *CA19-9* carbohydrate antigen 19-9, *AFP* alpha-fetoprotein, *ALB* albumin

### Features selected for the clinical model

**Fig. 2:**
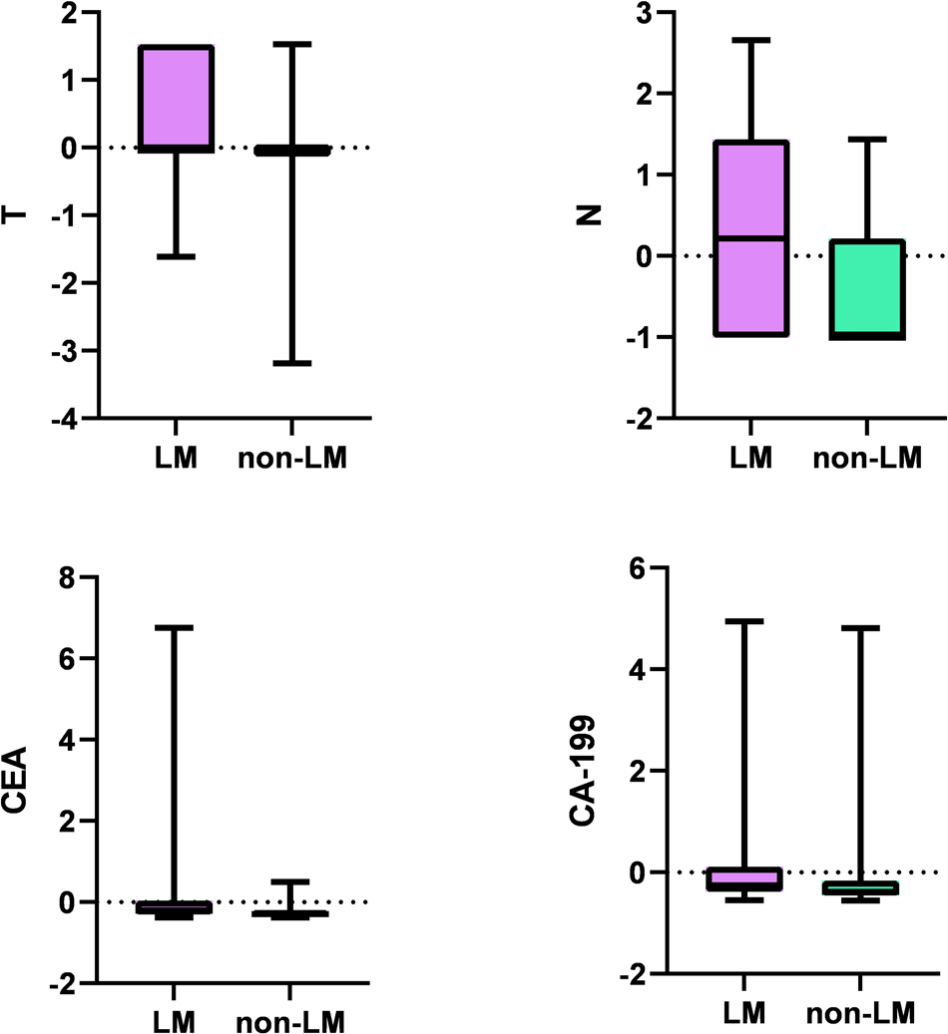
BOX plot of clinical features of CRLM and Non-CRLM sets

After *Z*-score normalization and *t*-test analysis, there were significant differences in clinical factors, including preoperative TNM stage and tumor markers (CEA and CA19-9), between patients with liver metastases from CRC (the CRCLM group) and those without (the non-CRCLM group) (*p* < 0.05). The distributions of these features in the CRCLM and non-CRCLM groups are shown in Fig. 2.

### Features selected for the CT radiomics model

**Fig. 3:**
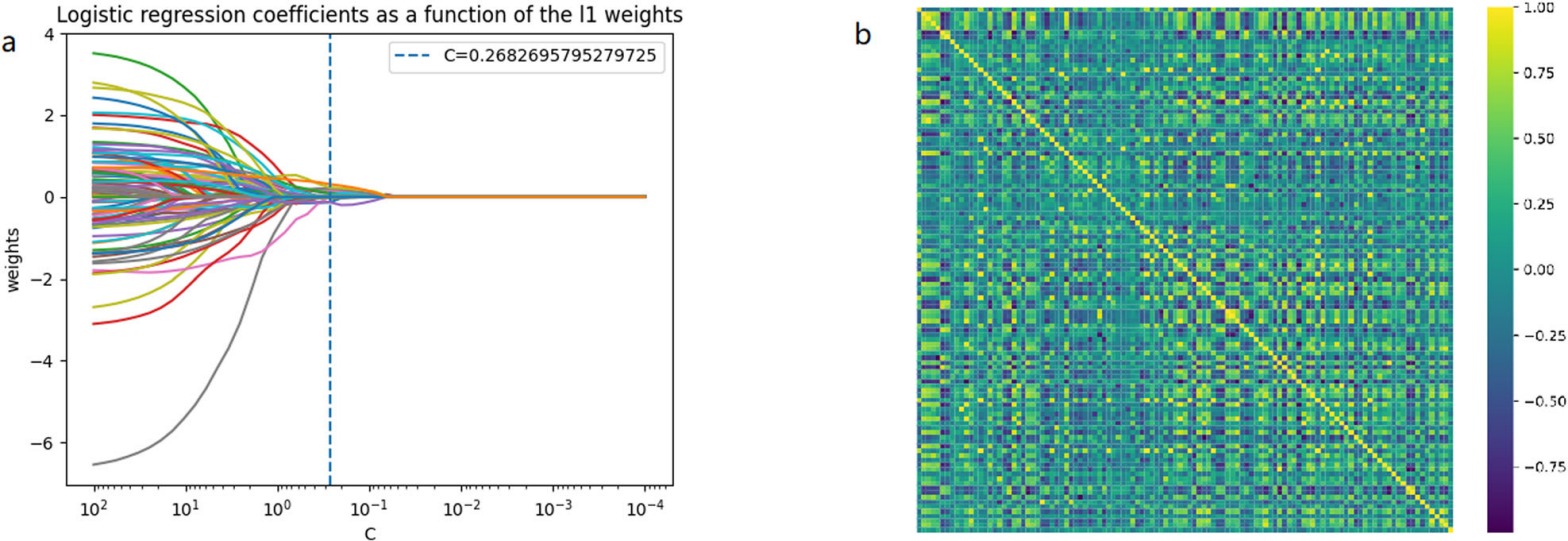
Feature selection process. Radiomics features were selected by the *t*-tests and L1 regularization based on logistic regression. **a** LASSO coefficient path diagram; **b** the feature heat map

**Fig. 4:**
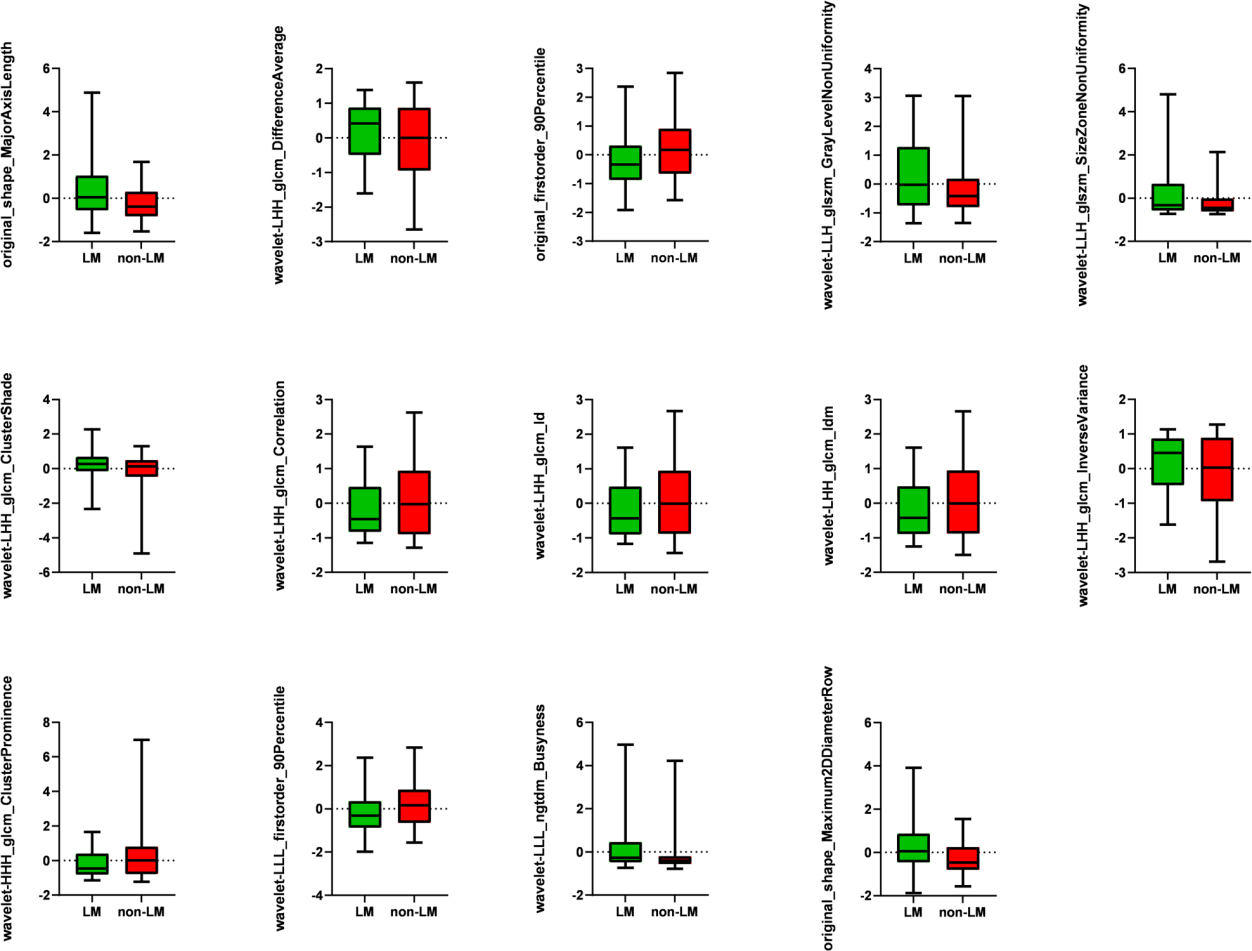
Box plot of radiomics features of CRLM and Non-CRLM sets

In total, 1051 radiomics features were computed, including original CT image features, wavelet image features, and Laplacian of Gaussian image features. Fourteen features were found to show statistically significant between-group differences (*p* < 0.05) by *t*-tests and least absolute shrinkage and selection operator (LASSO) analysis (L1 regularization). Figure 3a, b shows the feature heat map for LASSO analysis L1 regularization and *t*-test to select the best feature among all features, coefficient plot. The distributions of radiomics features in the CRCLM and non-CRCLM groups are shown in Fig. 4. The detailed Radiomics Features are presented in Table S1.

### Model validation and comparisons

We developed three pre-procedural diagnostic models for predicting liver metastases from CRC, namely, a clinical feature-based model, a radiomics feature-based model, and a fusion model. The radiomics feature-based model and clinical feature-based model showed performance in the validation set (with respective mean AUCs of 0.710 ± 0.065 and 0.713 ± 0.064). Using score-level methods, various features in the radiomics and clinical models were examined to identify which combination of features was more conducive to improving prediction performance. Ultimately, the fusion model using the 0.4*Rad + 0.6*Cli score fusion strategy achieved the best performance in the validation set (mean AUC 0.761 ± 0.060, 95% confidence interval: 0.644–0.878; *p* < 0.05). The performance of the various combinations of radiomics and clinical features for each of the models is described in detail in Table 2.

**Table 2:** The diagnostic performance of different combinations of radiomics and clinical features

Model	AUC	95% CI
Rad	0.710 ± 0.065	[0.584, 0.835]
Cli	0.713 ± 0.064	[0.589, 0.846]
Minimum	0.682 ± 0.068	[0.548, 0.815]
Maximum	0.741 ± 0.061	[0.624, 0.860]
0.1*Cli + 0.9*Rad	0.712 ± 0.064	[0.585, 0.838]
0.2*Cli + 0.8*Rad	0.738 ± 0.062	[0.616, 0.860]
0.3*Cli + 0.7*Rad	0.751 ± 0.060	[0.632, 0.865]
0.4*Cli + 0.6*Rad	0.756 ± 0.060	[0.638, 0.874]
0.5*Cli + 0.5*Rad	0.758 ± 0.060	[0.640, 0.876]
0.6*Cli + 0.4*Rad	0.761 ± 0.060	[0.644, 0.878]
0.7*Cli + 0.3*Rad	0.752 ± 0.060	[0.633, 0.870]
0.8*Cli + 0.2*Rad	0.742 ± 0.061	[0.622, 0.862]
0.9*Cli + 0.1*Rad	0.729 ± 0.852	[0.605, 0.852]

Table 3 shows the accuracy, recall, specificity, precision, negative predictive values (NPVs), and F1 for each model in the validation set. The results indicate that the fusion model was the best of all the models. However, it is pertinent to mention that the model’s sensitivity underwent a marginal decline. Nevertheless, the overall results demonstrate that the fusion model exhibits commendable performance in predicting liver metastases in colorectal cancer. These observations imply the potential of the fusion model to serve as a valuable asset for precise classification in this particular medical field.

**Table 3:** The accuracy, recall, specificity, precision, NPV, and F1 of the internal and external validation sets for each model

Model	Radiomics model	Clinical model	Fusion models
Accuracy, (%)	67.2	64.1	65.6
Recall, (%)	59.3	59.3	55.6
Specificity, (%)	73.0	67.6	73.0
Precision, (%)	61.5	57.1	60.0
NPV, (%)	71.1	69.4	69.2
F1, (%)	59.3	58.2	57.8

*NPV* negative predictive value

**Fig. 5:**
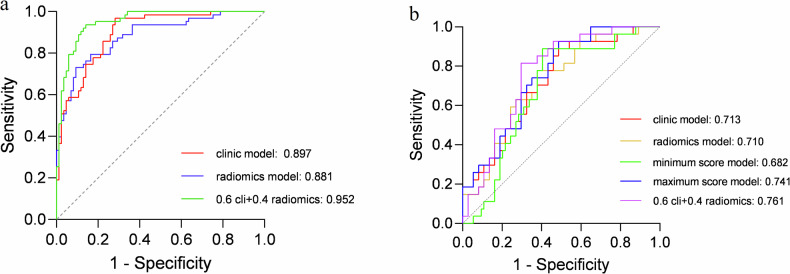
Comparison of receiver operating characteristic curve in a model trained with CRLM and different models. **a** Training set and (**b**) validation set. AUC, area under the receiver operating characteristic curve

Overall, the performance of the tumor features-based model was similar to that of the fusion model. The receiver-operating characteristic (ROC) curves for the three models are presented in Fig. 5a. The ROC curves for the three models in the training set are presented in Fig. 5b.

**Fig. 6:**
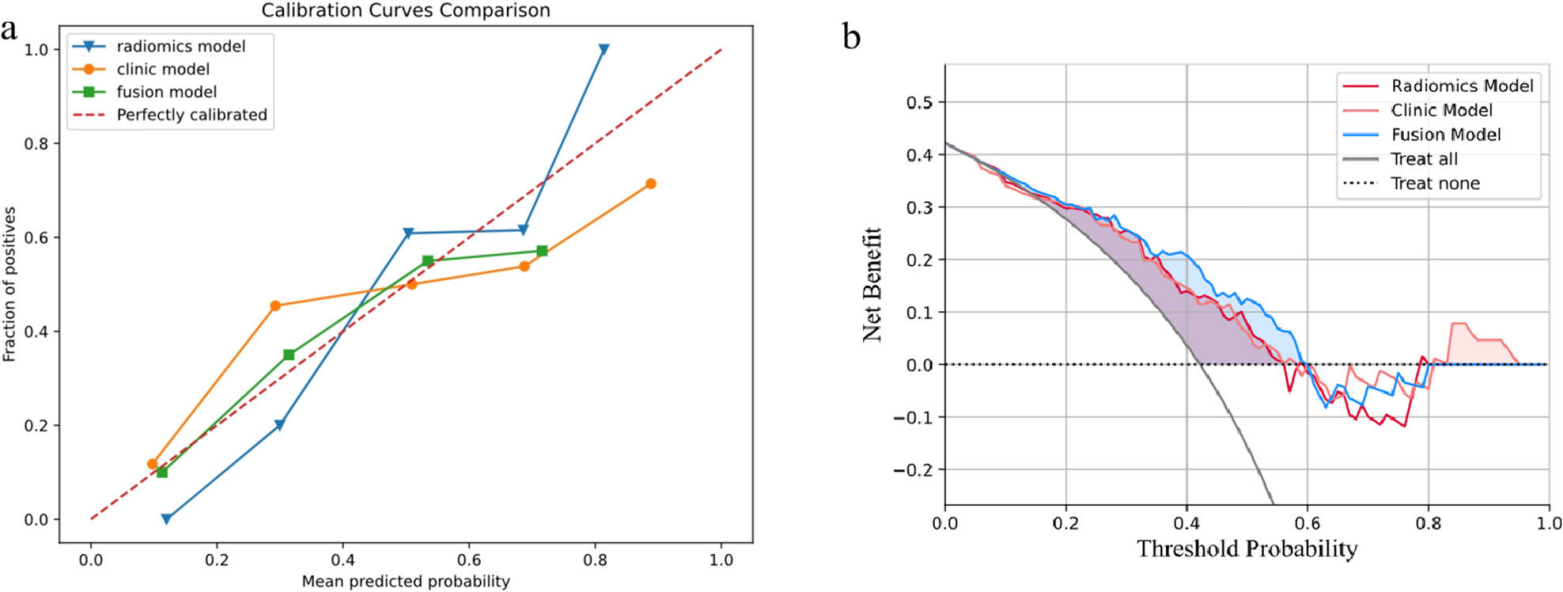
**a** Calibration curves of models in discriminating CRLM vs Non-CRLM; **b** DCA in using radiomics model, clinic model, and fusion model

Compared with the radiomics and clinical models, the fusion model showed better discrimination performance (Table 4). Calibration plots demonstrated good agreement between all the prediction models and actual observations of liver metastases from CRC (Fig. 6a). The decision curve analysis (DCA) plots showed that the net clinical benefit of the fusion model was better than that of the radiomics and clinical models (Fig. 6b). Furthermore, the calibration curves indicated a superior level of agreement.

**Table 4:** Comparison between models

Model	*Z*	*p*
Fusion model vs Rad	2.49	< 0.001
Fusion model vs Cli	2.86	< 0.001
Rad vs Cli	− 0.22	0.825

## Discussion

The liver is the organ most often affected by distant metastases of CRC, and many patients develop metastases despite successful resection. In this study, we evaluated a radiomics model that combined CT features and clinical data in the hope that it could be used as a non-invasive tool for the preoperative prediction of liver metastases after surgery for CRC. Our model showed good performance in both the training and validation sets (with respective AUCs of 0.952 and 0.761), indicating that the integration of radiomics features and clinical factors was satisfactory in all cohorts. In this model, tools that can confidently detect colorectal malignant metastases preoperatively may be valuable for patient management. Therefore, the imaging-clinicopathomics model has better performance and stability while processing different types of samples at different thresholds, while maintaining good sensitivity and accuracy [17].

Many researchers have investigated the relationship between imaging characteristics and tumor metastases. Studies by Lee et al and Taghavi et al have also shown that CT radiomics features and clinical features were effective in predicting liver metastases after surgery for CRC (with respective AUCs of 0.747 and 0.86). The study by Becker et al, which included 165 patients, found that whole-liver CT texture could predict patients at risk of developing liver metastases within ≤ 6 months (AUC 0.74) but not at later stages [18]. These findings suggest that metachronous metastases can develop in response to microenvironmental changes in patients with no apparent liver involvement at the time of diagnosis of CRC. Our studies have focused predominantly on CRC features rather than liver features, and some studies have shown that heterogeneity in primary CRC tumors is associated with the aggressiveness of liver metastases from CRC and can predict their likelihood. A combination of clinical features and radiomics features is useful for understanding the heterogeneity of colorectal tumors and their surrounding microenvironment. In view of the better diagnosis of colorectal cancer metastases, we emphasize the maximum stability and generalization ability, and more accurately diagnose the risk of missing potentially life-threatening diseases [17]. Among the various types of cancer, there is ample evidence that the metabolic state of tumors during metastasis is constantly adapting to the microenvironment [19, 20]. In studies by Liu et al and Shu et al, radiomics features obtained from magnetic resonance images of primary rectal tumors predicted synchronous liver metastases [21, 22].

Metachronous liver metastases significantly affect the prognosis of patients with CRC after radical colorectal resection. Previous studies have identified age, TNM stage, and tumor markers (CEA and CA19-9) to be clinical risk factors for colorectal metastasis [23–28]. Hao et al used clinical data to establish a novel nomogram, the AUC for which was 0.786, and the verification AUC was 0.784, which had a good differential effect on metachronous liver metastasis. Although these factors have good predictive performance, they cannot accurately determine the clinical stage or size of the negative tumor and the obsolescence of nomograms in radiomics research [29]. Other factors may also predispose to metachronous liver metastasis in CRC, including changes in the tissue microenvironment and tumor heterogeneity [30]. In our study, we used logistic regression and random forest classification, very common and widely studied machine learning models. For the prediction of liver metastases, the model trained by logistic regression showed the importance of adding imaging features to the clinical features compared with the ones only consisting of clinical features.

In this study, LASSO analysis (L1 regularization) and *t*-tests identified 14 signature radiomics features out of 1051 radiomics features, including three original image features and eleven wavelet image features. Tumor size is an important predictor of risk stratification in abdominal CT features. Maximum tumor diameter and shape were positively correlated with the risk of metastasis. First-order statistical characteristics reflect the degree of dispersion of gray values in the ROI. The higher the degree of malignancy of the tumor, the more the dispersion. Research by Cambria et al has shown that the potential for metastasis is closely related to the intratumoral environment [31]. Higher-order texture features reflect the uneven distribution of image texture in comprehensive information such as space and distance. This suggests that tumors with high malignant potential have complex internal structures and are more prone to heterogeneity.

This study has some strengths. First, unlike previous studies, it obtained clinical data that correlated with liver function and recurrence of CRC. Second, segmentation was based on the entire three-dimensional volume of the tumor, and radiomics features were extracted using a machine learning algorithm to maximize the potential information underlying the images and identify features with the highest predictive value. Random forests can process high-latitude data without feature selection, are strongly resistant to overfitting, and can detect interaction between features.

There are also some limitations to our study. First, it had a retrospective single-center design. A prospective multicenter study is required in the future. Second, as a retrospective study with a limited data set, selection bias and the presence of unknown confounders were possible. Although we tried to minimize selection bias by using rigorous inclusion criteria, we could not rule out unknown confounders that could have affected the results. Third, in our study, the sample was predominantly from the same region, and this imbalance in demographic and geographic distribution may have limited the model’s understanding of population characteristics such as different regions and ethnicities. To mitigate the impact of this potential bias, we will employ a broader sample selection strategy in future studies to ensure that different regions and population groups are adequately represented.

In conclusion, this study found that a model that included CT radiomic-based features and clinical features using the RL algorithm was better able to predict liver metastases in patients with CRC than a radiomics model or clinical model alone.

## Supplementary information

ELECTRONIC SUPPLEMENTARY MATERIAL

## Abbreviations

AFP: Alpha-fetoprotein
ALB: Albumin
AUC: Area under the curve
BMI: Body mass index
CA19-9: Carbohydrate antigen 19-9
CEA: Carcinoembryonic antigen
CT: Computed tomography
DCA: Decision curve analysis
LASSO: Least absolute shrinkage and selection operator
NPV: Negative predictive value
ROC: Receiver-operating characteristic
ROI: Region of interest
