# Supplementary material for: Development and validation of a radiopathomics model for predicting liver metastases of colorectal cancer
Source: Eur Radiol. 2024 Dec 2;35(6):3409–17. doi: 10.1007/s00330-024-11198-1 (PMC12081500; doi:10.1007/s00330-024-11198-1)
Supplement: Supplementary file 1 — ELECTRONIC SUPPLEMENTARY MATERIAL [file 330_2024_11198_MOESM1_ESM.pdf]

Development and validation of a radiopathomics model for predicting liver metastases of colorectal cancer

ELECTRONIC SUPPLEMENTARY MATERIAL

Figure S1. The flowchart of our proposed prediction model.

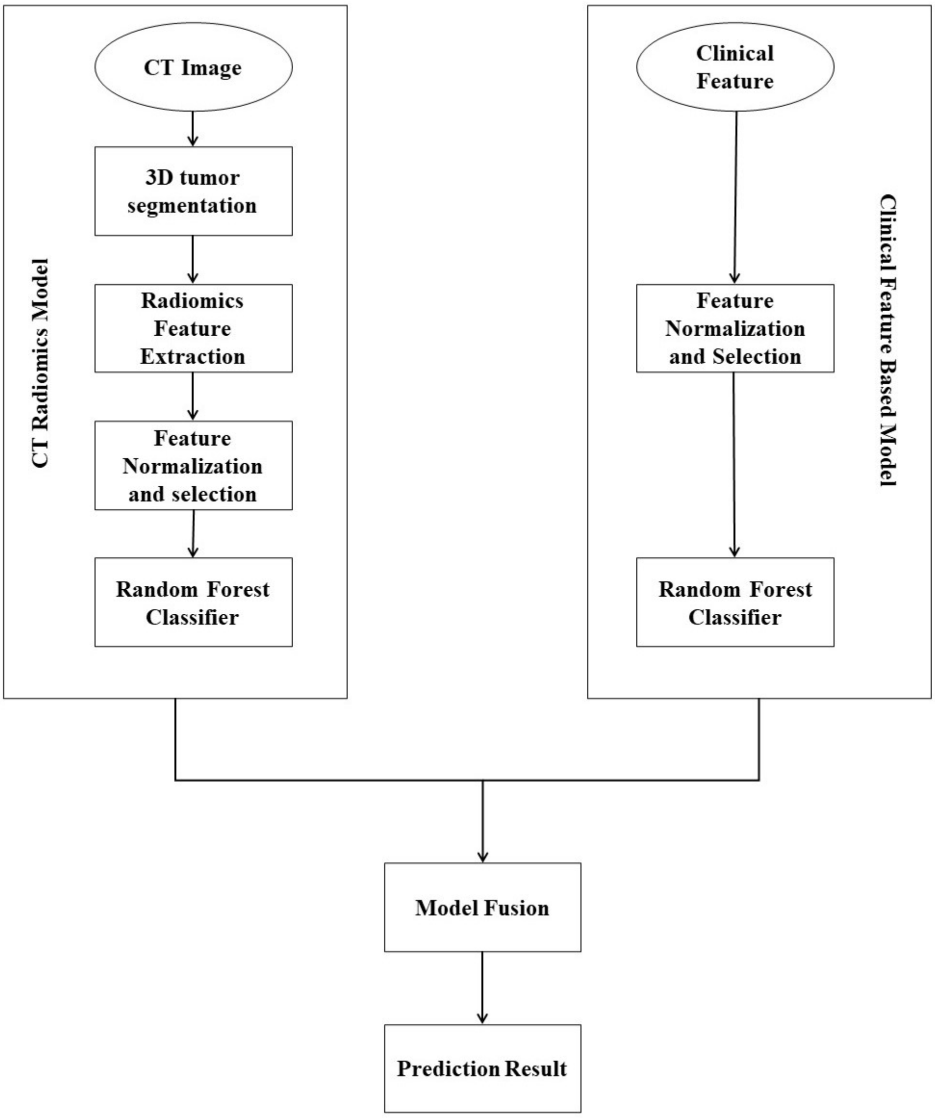

Table S1 Detailed Radiomics Features

Radiomics Features	Original	Wavelet
		LLH_glszm_GrayLevelNonUniformity
	shape_Maximum2DDiameterRow	LLH_glszm_SizeZoneNonUniformity
		LHH_glcm_ClusterShade
		LHH_glcm_Correlation
	shape_MajorAxisLength	LHH_glcm_DifferenceAverage
		LHH_glcm_Id
		LHH_glcm_Idm
		LHH_glcm_InverseVariance
	firstorder_90Percentile	HHH_glcm_ClusterProminence
		LLL_firstorder_90Percentile
		LLL_ngtdm_Busyness
